# On the Use of Low-Cost Radar Networks for Collision Warning Systems Aboard Dumpers

**DOI:** 10.3390/s140303921

**Published:** 2014-02-26

**Authors:** José-Tomás González-Partida, Francisco León-Infante, Rodrigo Blázquez-García, Mateo Burgos-García

**Affiliations:** ETSI de Telecomunicación. Departamento de Señales, Sistemas y Radiocomunicaciones. Universidad Politécnica de Madrid, Av. Complutense s/n, Madrid 28040, Spain; E-Mails: francisco.leon.infante@alumnos.upm.es (F.L.-I.); rodrigo.blazquez.garcia@alumnos.upm.es (R.B.-G.); mateo@gmr.ssr.upm.es (M.B.-G.)

**Keywords:** radar, interference, clutter, network, collision warning, dumper, low-cost

## Abstract

The use of dumpers is one of the main causes of accidents in construction sites, many of them with fatal consequences. These kinds of work machines have many blind angles that complicate the driving task due to their large size and volume. To guarantee safety conditions is necessary to use automatic aid systems that can detect and locate the different objects and people in a work area. One promising solution is a radar network based on low-cost radar transceivers aboard the dumper. The complete system is specified to operate with a very low false alarm rate to avoid unnecessary stops of the dumper that reduce its productivity. The main sources of false alarm are the heavy ground clutter, and the interferences between the radars of the network. This article analyses the clutter for LFM signaling and proposes the use of Offset Linear Frequency Modulated Continuous Wave (OLFM-CW) as radar signal. This kind of waveform can be optimized to reject clutter and self-interferences. Jointly, a data fusion chain could be used to reduce the false alarm rate of the complete radar network. A real experiment is shown to demonstrate the feasibility of the proposed system.

## 1. Introduction

Maneuvering dumpers on building sites are dangerous for people and vehicles working near them. The large dimensions and design of dumpers produce many blind zones for the machine operators that complicate their driving tasks. In order to reduce the number of accidents, a collision warning system must be designed and implemented. To cover the whole perimeter of the dumper, several sensors working jointly will be required. Furthermore, sensor redundancy will help to reduce the number of false alarms.

Available sensors for this kind of systems could be radars, ultrasonics and optronics. The good performance of radar sensors under adverse conditions like dust, rain, fog, dark, *etc*. and their large coverage make this sensor a good candidate for a warning collision system. Nowadays, commercial low-cost radar transceivers, operating in X and K-band, are available, and some of them have enough bandwidth to achieve the range resolution lower than 1 m that would be desired for this application [1]. This is the case of the radar transceivers produced by companies like RFbeam Microwave GmbH (St. Gallen, Switzerland), Microwave Solution Ltd. (Hemel Hempstead, UK), Agilsense ST Electronics (Singapore), Smart Microwave Sensors GmbH (Braunschweig, Germany), *etc*.

**Figure 1.**
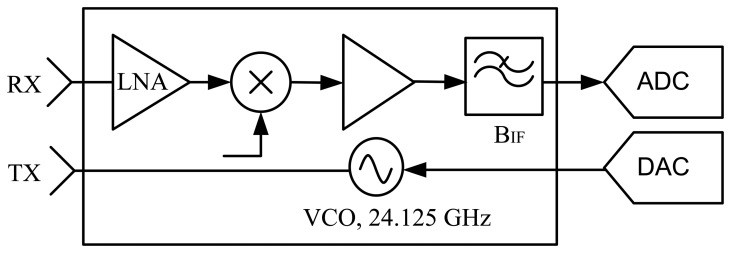
Linear Frequency Modulated Continuous Wave (LFM-CW) low-cost radar sensor.

Here, we propose the design of a radar network, using low-cost Linear Frequency Modulated (LFM) transceivers operating in the K-band. The block diagram of a transceiver is depicted in Figure 1.

The use of radar networks in automotive applications has been extensively discussed in the literature [2–5]. However, for this application, it is very important to work with a very low false alarm rate. On one hand, stopping a dumper frequently due to false alarms reduces the productivity of these machines, and it is not a cost effective solution. On the other hand, dumper operators must trust in the system. A collision warning system that generates several false alarms in a working day may be discredited by the dumper driver, with fatal consequences when a real detection is ignored.

The main sources of false alarms in this application are the surface clutter and the interferences of other radar sensors of the network. The second section of this paper analyzes the ground clutter problem from the point of view of the signal processing of LFM radars. Furthermore, antenna location and beamwidth are discussed in this section. The third section studies different alternatives to avoid interferences between the radars of the network. Finally, a novel waveform, called Offset LFM-CW (OLFM-CW), is proposed. This solution maintains the hardware simplicity and performance of an isolated LFM-CW sensor.

Finally, in the fourth section, experimental results with the proposed sensor network are presented. This experiment demonstrates the correct performance of a radar network operating with OLFM-CW signals.

## 2. Ground Clutter with LFM Sensors

**Figure 2.**
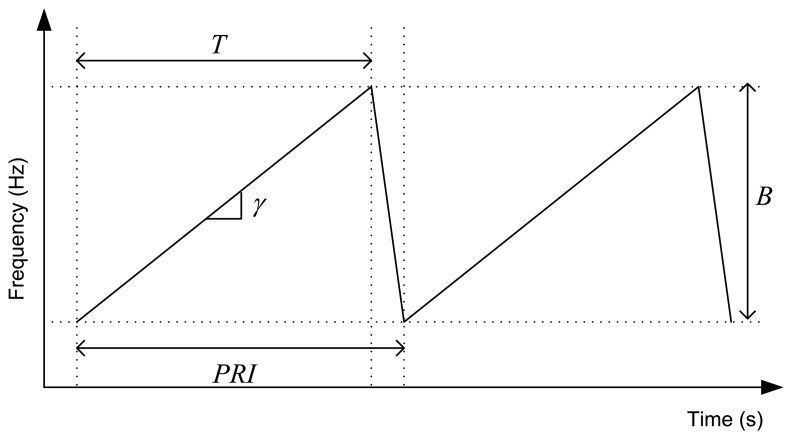
LFM-CW waveform.

LFM radars transmit a burst of frequency ramps as shown in Figure 2. Each received ramp is demodulated with a copy of the transmitted ramp, like the scheme of Figure 1 depicts. A Fast Fourier Transform (FFT) of the received signal performs the pulse compression, and the range profiles are obtained [6,7]. Then, we can allocate several consecutives range profiles in a matrix form, and carry out a FFT across them. In this way, the Doppler compression is achieved, and a range-Doppler map is obtained [6,7]. Only the ascending ramp is processed. The descending ramp, that usually represents the 10% of the duty cycle, is necessary due to practical hardware implementation.

Ground target detection at short ranges is mainly limited by surface clutter. Typically, LFM systems carry out the detection task over the range-Doppler domain, because a higher signal-to-clutter ratio can be achieved using this domain. Therefore, it is interesting to analyze how the surface clutter is mapped into this detection domain and how a right selection of the antenna location, the antenna beamwidth, and the signal parameters can improve the detection performance.

### 2.1. Range-Doppler Mapping of Surface Clutter

**Figure 3.**
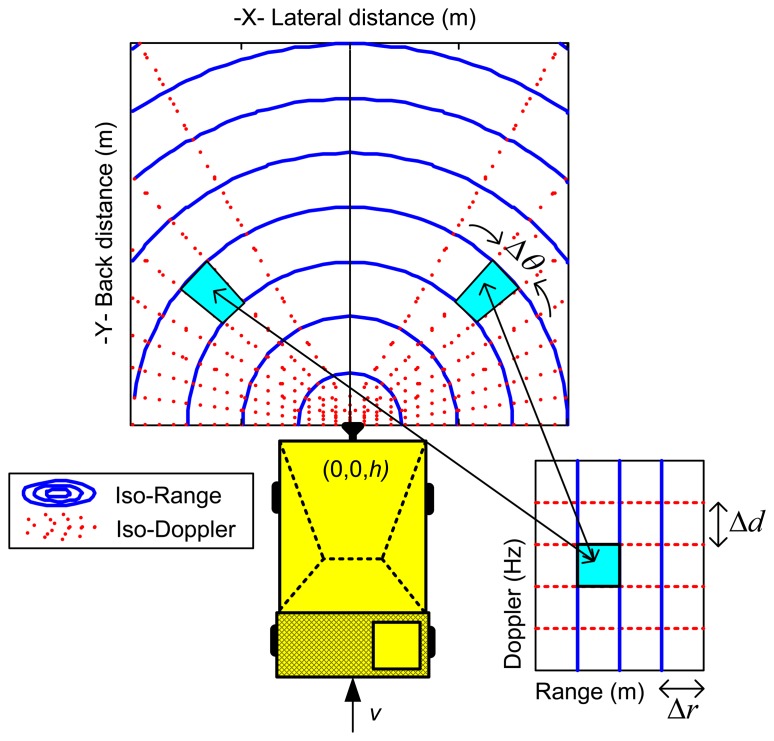
Range-Doppler mapping of the ground surface.

Suppose a radar is located at a height *h* aboard a dumper that moves at constant velocity *v* on an almost flat terrain. Two different points of the terrain will be mapped into the same point of the range-Doppler domain. This mapping is well known to the synthetic aperture radar (SAR) community. The flat terrain can be divided using iso-Range and iso-Doppler curves. Points of the terrain that exhibits the same range and Doppler values are obtained from the intersection of the ground plane, a range sphere, and a Doppler cone [8,9]. Figure 3 shows the iso-curves projection on the ground plane.

For each point of the range-Doppler domain there are two points at the ground surface producing an echo with these range and Doppler values. In practice, the range-Doppler map is divided into rectangular range-Doppler cells due to the limited resolution of the radar system. The size of the cells is equal, and is determined by the range (Δr) and Doppler (Δd) resolution of the radar system. The clutter contribution to a range-Doppler cell is the sum of the contributions of two symmetrical zones of the terrain surface. Figure 3 illustrates this assignment.

The radar cross section (RCS), *σ*, of the clutter that is integrated within a range-Doppler cell can be estimated computing the area of the two symmetrical zones *A_c_*, and multiplying it by the ground reflectivity *σ_0_* (m^2^/m^2^):

(1) $$\sigma =A_{c}\cdot \sigma_{0}.$$

There is a trade-off between antenna beamwidth and antenna gain. A large azimuth antenna beamwidth is desired to enlarge the field of view of the surveillance area, however, the antenna gain would be lower and the detection capability would be reduced. Elevation antenna beamwidth should be narrow in order to attenuate the nearest ground reflections. Nevertheless, this cannot be too narrow because targets at ground level, e.g., a lying person, could be missed. The optimal solution depends on the specific radar and antenna. The values of our particular system are specified in Table 1.

Using a radar network a broader coverage in azimuth can be achieved. Furthermore, false alarm reduction and angle determination could be addressed using data fusion [3,9].

Ground reflectivity, *σ_0_*, depends on the grazing angle; the larger the grazing angle, the larger the reflectivity of the terrain [10]. In order to reduce the grazing angle, and consequently the ground reflectivity *σ_0_*, the radar sensors should be located not too much high respect to the ground level. However, the height *h* must not be too much low, since the nearest ground reflections would be received by the main lobe of the antenna beam.

Typically, *h* value oscillates between 1 and 2 m. For these low heights, the clutter area that is integrated within a range-Doppler cell of central coordinates (*r_j_*, *d_j_*) can be approximated by the sum of two equal sectors of circular crown:

(2) $$A_{c}=2\cdot r_{j}\cdot \Delta r\cdot \Delta \theta$$

where *Δθ* is obtained with Equations (3) and (4):

(3) $$\Delta \theta =2\cdot \sin^{-1}\left(\frac{\lambda \cdot \Delta d}{4\cdot v\cdot \sin\left(\theta_{j}\right)}\right)$$

(4) $$\theta_{j}=\cos^{-1}\left(\frac{\lambda \cdot d_{j}}{2\cdot v}\right)$$

Being *λ* the wavelength of the radar and *v* the velocity of the dumper.

**Figure 4.**
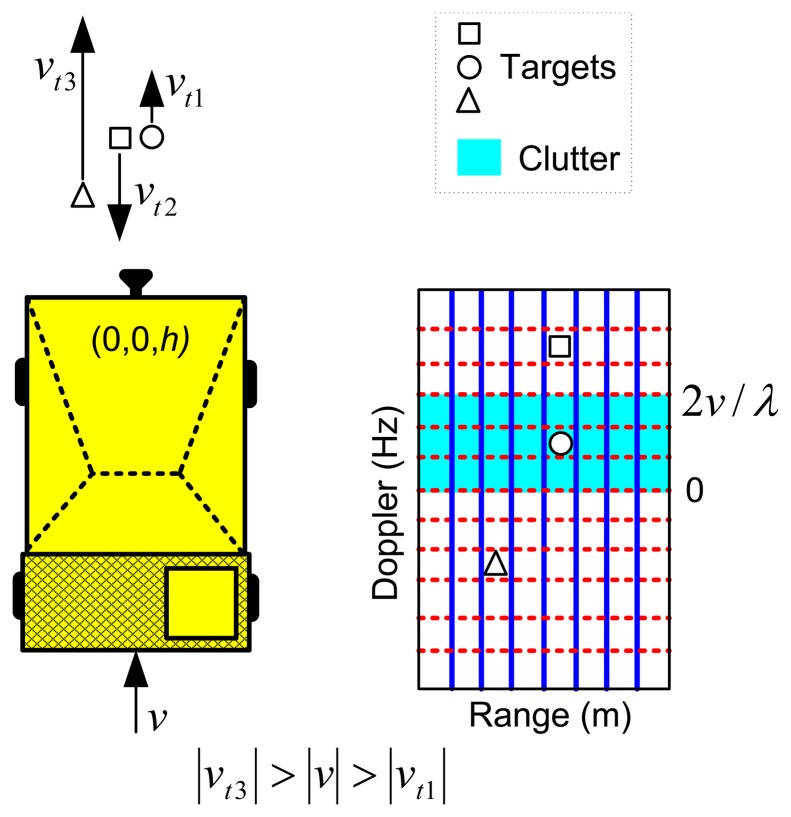
Dependencies of target and dumper velocities in the clutter mapping.

The velocity of the dumper has influence on the distribution of the power clutter within the range-Doppler map. Suppose that a fixed PRF has been selected to avoid Doppler aliasing. For a fixed Coherent Processing Interval (CPI), i.e., a fixed Doppler resolution, Equations (2) and (3) demonstrate that a high velocity of the dumper spreads the clutter energy over more range-Doppler cells, but the integrated area of clutter at each cell is reduced. However, with a low velocity of the dumper, the number of range-Doppler cells affected by the clutter is reduced, but the integrated area of clutter at each cell is increased. This phenomenon is depicted in Figure 4.

In the case of a stopped dumper or with a speed lower than the velocity resolution, Equation (3) should be replaced by *Δθ* = *θ_a_*, i.e., the azimuth antenna beamwidth. This means that all the ground clutter is integrated in the zero-Doppler row of the range-Doppler map.

The speed of the target, *v_t_*, has also influences in the detection performance. A target coming to the radar falls on the free clutter zone of the range-Doppler domain. A stopped target or a target going away to the radar with a velocity lower than the dumper falls on the clutter zone. A target going away to the radar with a velocity higher than the dumper falls on the free clutter zone.

## 2.2. Parameter Selection for LFM-CW Radar

Equations (2) and (3) demonstrate that improving the range and Doppler resolution, the clutter area integrated in a range–Doppler cell is reduced, and thus the clutter RCS given by Equation (1).

For collision warning applications an update rate up to 100 ms is usually specified [1]. Therefore, this would be the maximum CPI. Using commercial sensors in K-band, with an available bandwidth of *B* = 250 MHz, we can obtain a maximum range resolution of *c*/2*B* = 0.6 m and a maximum Doppler resolution of CPI^−1^ = 10 Hz, i.e., a velocity resolution of 0.23 km/h.

The Pulse Repetition Interval (*PRI*) must be chosen to avoid Doppler ambiguities. If the maximum expected velocity of the dumper would be *v* = 35 km/h and for the target *v_t_* = 15 km/h. The worst case gives a maximum velocity of *v_max_* = *v* + *v_t_* = 50 km/h. Therefore, the *PRI* to avoid ambiguities in Doppler would be approximately 200 μs, accordingly with Equation (5):

(5) $$\text{PRI}\leq \frac{\lambda }{4\cdot v_{\max}}$$

To achieve the desired velocity resolution of 0.23 km/h, a FFT Doppler processing using CPI/*PRI* = 500 consecutive ramps is necessary. Finally, the IF bandwidth must be chosen taking into account the maximum range to be explored by the radar, *R_max_*. Using Equation (6), for *R_max_* = 30 m, the IF bandwidth would be *B_IF_* ≥ 278 kHz:

(6) $$B_{IF}\geq \frac{2\cdot R_{\max}}{c}\cdot \gamma =\frac{2\cdot R_{\max}}{c}\cdot \frac{B}{T}$$

where *c* is the light velocity, *γ* is the chirp rate, *B* = 250 MHz is the sweep bandwidth, and *T* = 180 μ*s* is the ascending ramp duration.

### 2.3. Detection Requirements

Due to the application, the complete network has been specified with a very low false alarm rate, i.e., one false alarm per workday (8 h). The detection probability will be the highest that can be obtained with this false alarm constraint. This way, the system should detect a person with a probability higher than 0.95, false alarm probability lower than 10^−1^°, with a low transmitted power, and in a ground clutter environment. This is the challenge of this system.

**Figure 5.**
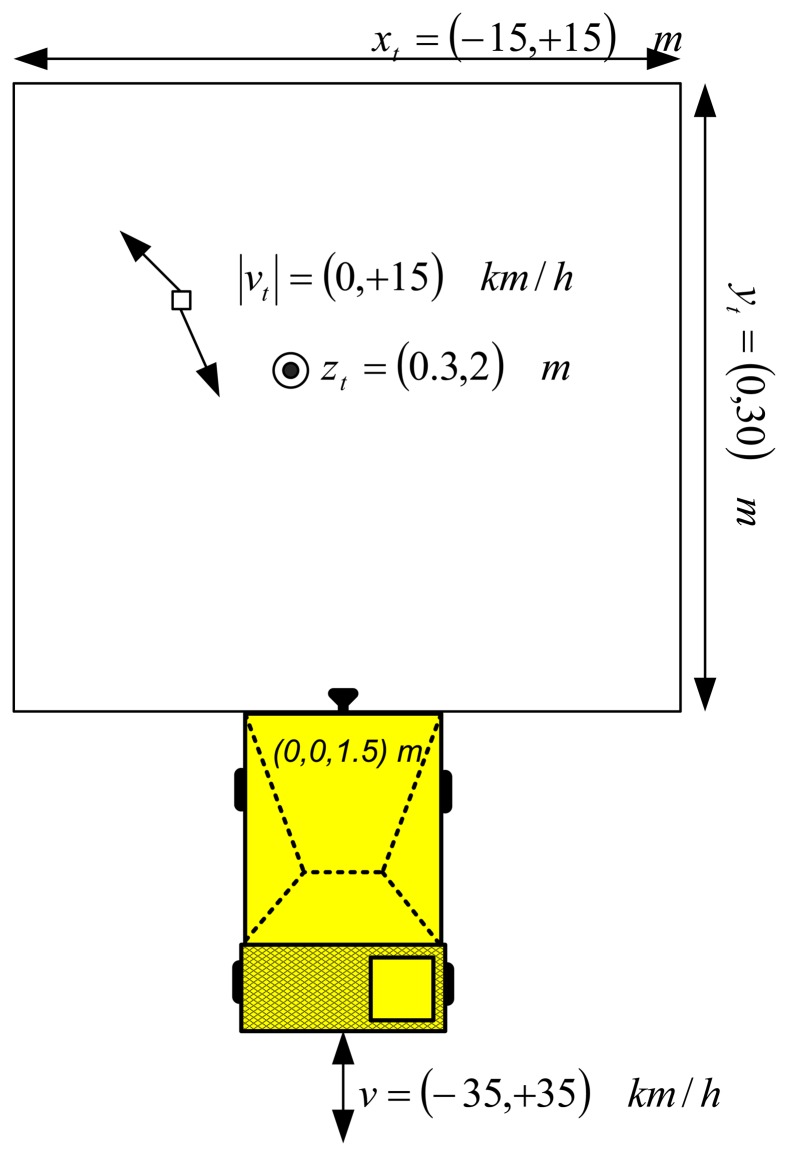
Scheme of the Monte Carlo simulation.

With the parameters derived in Section 2.2, Monte Carlo simulations have been done in order to estimate the detection probability and false alarm rate of a single radar sensor in a ground clutter environment. The simulation procedure consists of modeling a person as a Swerling 1 target, with RCS of 1 m^2^. The position and velocity of the target has randomly selected for each Monte Carlo iteration. Also the velocity of the dumper has been randomly chosen. The scheme of the Monte Carlo simulation, with the possible random values, is illustrated at Figure 5.

On one hand, the ground clutter model used in our simulations is the GIT's model [10] for soil/sand terrain. GIT's model provides expected values of σ_0_ for different terrains, grazing angles and frequencies. Other σ_0_ models for ground clutter are Morchin's model and Gamma model [10].

On the other hand, people and vehicles are the main targets that must be detected by the system. This way, the dynamic range of the system should cover their detection. RCS of these targets ranges from 1 m^2^ (pedestrian) to 200 m^2^ (large vehicles).

**Figure 6.**
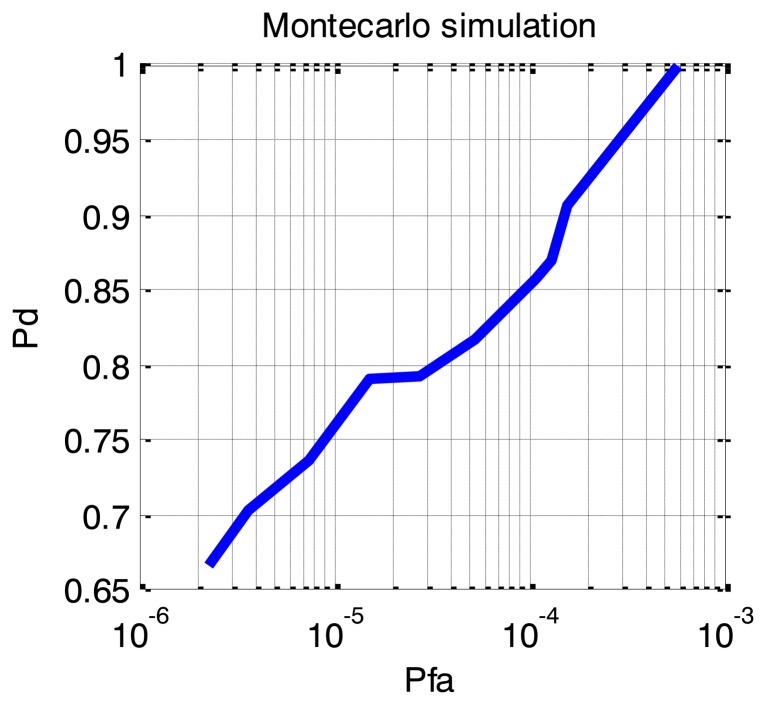
Detection performance of a single sensor. Monte Carlo simulation.

For the detection task and adaptive detector, called OSCA-CFAR, has been used. This is a bi-dimensional combination of Order Statistic (OS) and Cell Averaging (CA) CFAR detectors [11]. OSCA-CFAR has been specifically designed to work in multi-target situations within range-Doppler maps. This detector has been chosen due to its suitability to detect moving persons with extended Doppler signatures. The threshold of the detector has been iteratively changed to obtain the P_d_-P_fa_ (Detection *vs*. false alarm probability) curve shown in Figure 6.

Simulation results show that a single sensor cannot meet the detection requirements for this application. To fulfill these requirements a radar network has to be used for improving the performance of a single sensor. Using a radar network with data fusion and tracking, the number of false tracks can be reduced although the false alarm rate of the individual radars may be high. The suppression of single sensor false alarms by the data fusion allows a reduction of detection thresholds within the separate sensor detection algorithms. The result is an increase in sensitivity when using a sensor network compared with using a single sensor [3].

## 3. Radar Network Self-Interferences

The previous section analyzes the operation of a single radar sensor; however the proposed system is a combination of several radars forming a network. All the sensors should guarantee an update rate of up to 100 ms. Therefore, there is a challenge to share the resources of time and frequency between the *n* sensors of the network, avoiding interferences between them in order to maintain a low false alarm rate [12,13].

Use of LFM-CW signals with random variations on signal parameters, e.g., delay, *PRI*, bandwidth, chirp rate, have been analyzed in the literature to overcome the interferences between LFM-CW systems [14]. However, this solution only minimizes the effects of the interferences, while some of them remain. Therefore, a solution that completely removes the interferences is preferable. In the following sections different alternatives for working in our network with *n* radar sensors without self-interferences are analyzed.

### 3.1. FDMA (LFM-CW)

**Figure 7.**
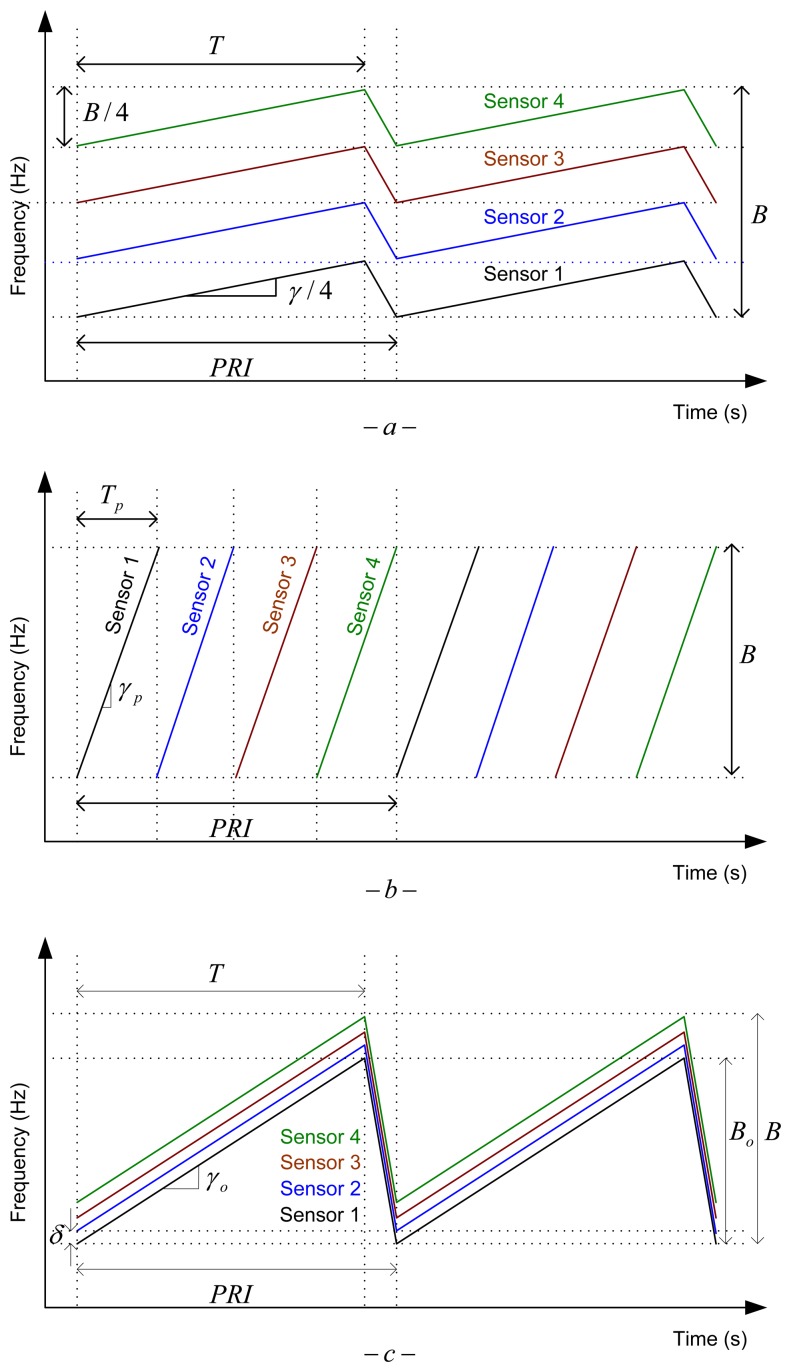
LFM waveforms: (**a**) FDMA LFM-CW (**b**) TDMA pulsed-LFM (**c**) Offset LFM-CW.

First solution consists in dividing the available bandwidth *B* between the *n* sensors in a Frequency Division Multiplex Access (FDMA) philosophy. An example for *n* = 4 is shown in Figure 7a.

In this configuration, each sensor uses a LFM-CW signal with the same *PRI* and ramp duration *T* as in the single sensor case, but with a reduced bandwidth of *B*/*n*. Consequently, range resolution is worsen by a factor *n*. This is not a suitable solution for our application that needs good resolution and range accuracy on short distances. Furthermore, the ground clutter area integrated in each range-Doppler cell is increased due to the reduction of range resolution.

### 3.2. TDMA (LFM Pulses)

Other solution is to divide the available time *PRI* between the *n* sensors in a Time Division Multiplex Access (TDMA) philosophy. An example for *n* = 4 is illustrated in Figure 7b. This way, each sensor uses LFM pulses with the same bandwidth *B* and *PRI* as in the single sensor case, but with a lower ramp duration *T_p_* = *PRI*/*n* < *T*. This reduction of the ramp duration supposes a reduction by a factor *n* of the average transmitted power for each sensor. Again, this is not a suitable solution for our application, because the sensitivity of the low-cost transceivers sensors is typically low, and a reduction of the Signal- to-Noise Ratio (SNR) would not be acceptable to fulfill the maximum range requirement. Furthermore, the pulsed LFM scheme needs a synchronization subsystem that complicates the signal generation and acquisition hardware, and increases the cost of the system. The last disadvantage is that the IF bandwidth and the sampling frequency of the Analog-to-Digital Converter (ADC) must be increased by a factor *n*, approximately, increasing the complexity of the ADC and the radar signal processor.

### 3.3. Offset LFM-CW

A novel alternative suitable for this kind of short range sensors is the use of Offset LFM-CW signals. The idea is to generate LFM-CW signals with a constant frequency offset *δ* between consecutives sensors of the network. An example is depicted in Figure 7c for *n* = 4.

With this solution each sensor uses a LFM-CW signal with the same *PRI* and ramp duration *T*, as in the single sensor case, but with a bandwidth a bit lower *B_o_* < *B* than in the single sensor situation. To avoid interferences between the different sensors the frequency offset *δ* must be chosen taking into account the expected maximum detectable range of a sensor, *R_sen_*, and the available IF bandwidth Equation (7):

(7) $$\delta \geq \frac{2\cdot R_{\text{sen}}}{c}\cdot \frac{B_{o}}{T}B_{IF}$$

where the sweep bandwidth of each signal has been reduced to *B_o_*:

(8) $$B_{o}=B-(n-1)\cdot \delta (8)$$

Combining expressions (7) and (8), the necessary offset can be determined using Equation (9):

(9) $$\delta \geq \frac{2\cdot R_{\text{sen}}\cdot B+c\cdot T\cdot B_{IF}}{2\cdot R_{\text{sen}}\cdot (n-1)+c\cdot T}$$

This frequency offset assures that the beat signal resulting from mixing the transmitted signals of two different sensors has a beat frequency higher than the cut-off frequency of the low-pass IF filter. Also, the signal transmitted by a sensor, reflected by a target at a distance lower than *R_sen_*, and demodulated with the transmitted signal of a different sensor, has a beat frequency that is rejected by the IF filter.

This way, all the self-interferences will be rejected by the IF filter of each sensor. The range resolution of each sensor would be slightly worse than in the single sensor situation, but this reduction is very low and the requirements will be still met. E.g., suppose a typical system with a *PRI* = 200 μs, *T* = 180 μs, *B* = 250 MHz, *R_sen_* = 100 m, *B_IF_* = 300 kHz. Using Equations (8) and (9) we obtain *δ* = 1.21 MHz and *B_o_* = 246.37 MHz. This means a reduction of the range resolution of 1.5% that is negligible for this application.

This solution removes the interferences completely, and guarantees the same detection performance as in the single sensor situation. Furthermore, the generation and acquisition subsystem are the same as in the case of a single LFM-CW sensor. Only a different frequency offset must be introduced in the signal of each sensor. This frequency offset means a simple voltage offset at the output of the Digital-to-Analog Converter (DAC) for the sensor scheme of Figure 1. This signal configuration has all the advantages of LFM-CW systems: hardware simplicity, low peak power transmitted, and high range and Doppler resolution.

**Figure 8.**
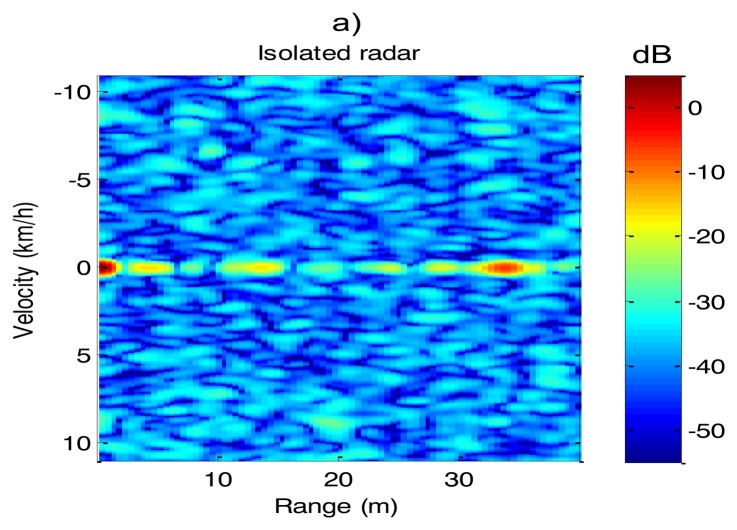

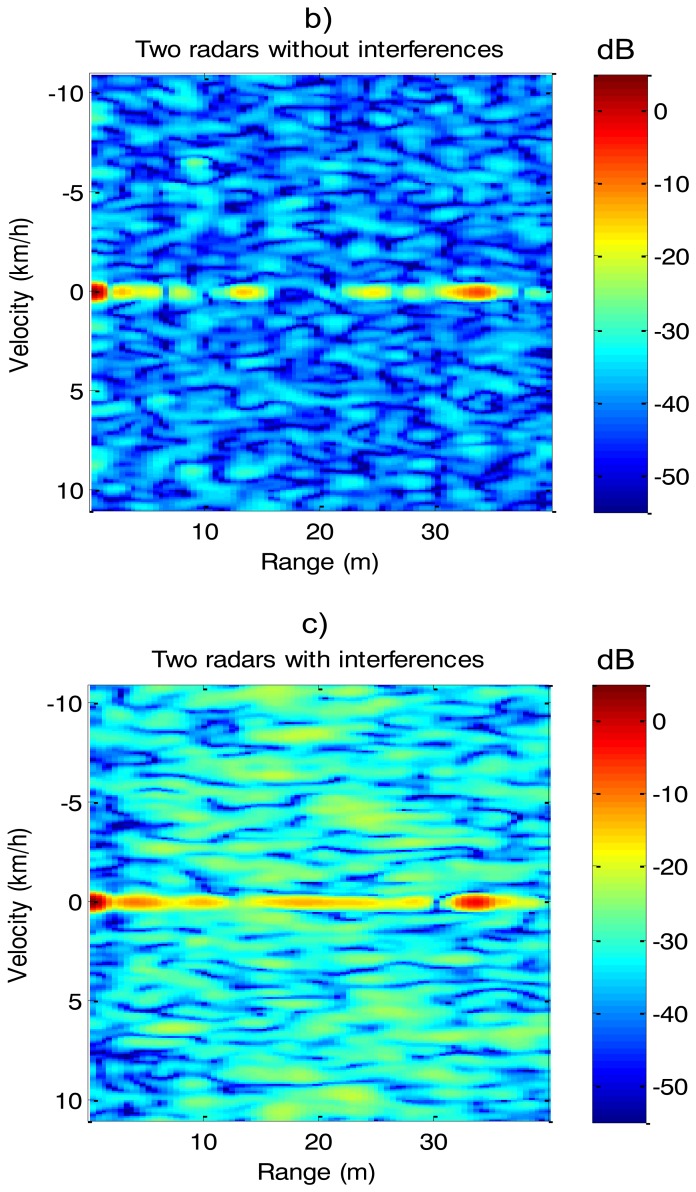
Range-Velocity maps (**a**) single radar (**b**) two non-interfering radars (**c**) two interfering radars.

A real experiment with two radar sensors has been carried out to demonstrate the effectiveness of the interference suppression based on OLFM-CW waveform. Radars are configured with a *PRI* = 1.02 ms, *T* = 0.918 ms, *B* = 225 MHz, *R_sen_* = 100 m, *B_IF_* = 70 kHz. Using Equations (8) and (9) we obtain δ = 236 kHz and *B_o_* = 224.76 MHz. The experiment has been carried out in a corridor without moving targets. Results are illustrated at Figure 8:

Figure 8a shows a range-Doppler map of the corridor obtained from the first sensor when the second radar is turned off. This range-Doppler map will be used as reference. Figure 8b shows a range-Doppler map of the same corridor, adquired from the first sensor when the second sensor is turned on, but fulfilling the minimum frequency offset *δ* = 236 kHz. We can observe that the first sensor is working free of interferences comparing the similarity of Figures 8a,b.

Figure 8c shows a range-Doppler map of the same corridor, obtained from the first sensor when the second radar is turned on, but without fulfilling the minimum frequency offset δ = 236 kHz. This figure shows how the interferences have increased the noise floor, reducing the sensitivity of the first radar.

## 4. Experimental Results

**Figure 9.**
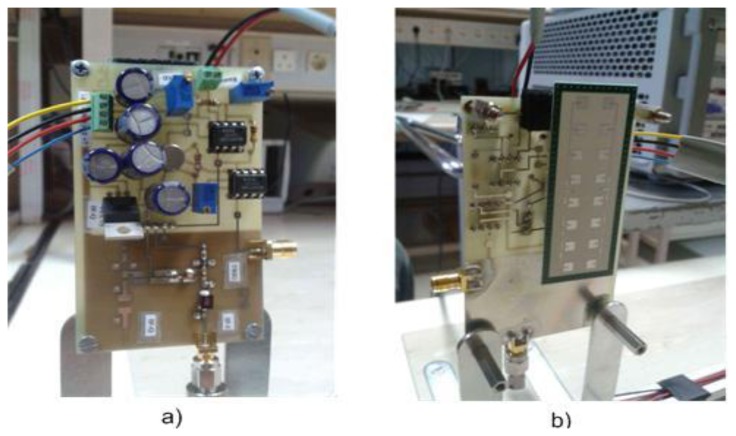

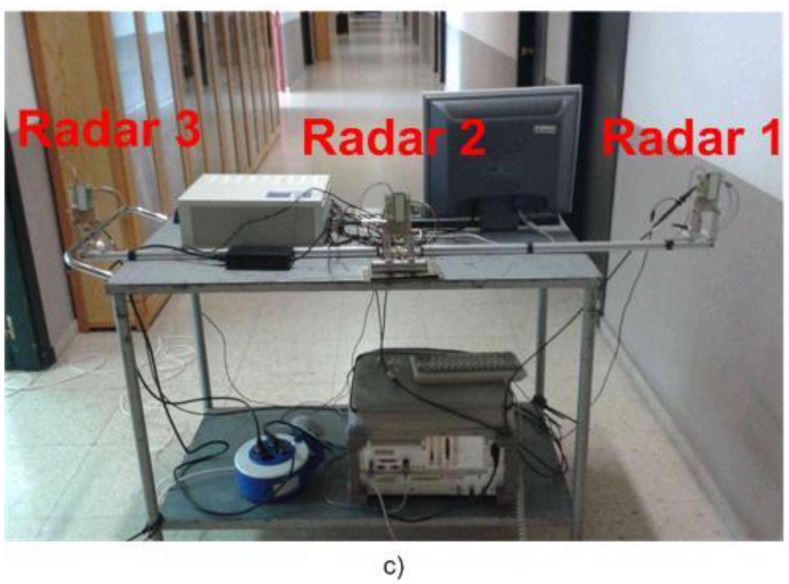
Radar network: (**a**) Back-view single sensor (**b**) Front-view single sensor (**c**) Radar network with three sensors.

A complete radar network with three sensors has been designed and developed. A photograph of the sensors and the radar network assembly is shown in Figure 9. Radar sensors have been configured with the parameters listed in Table 1.

**Figure 10.**
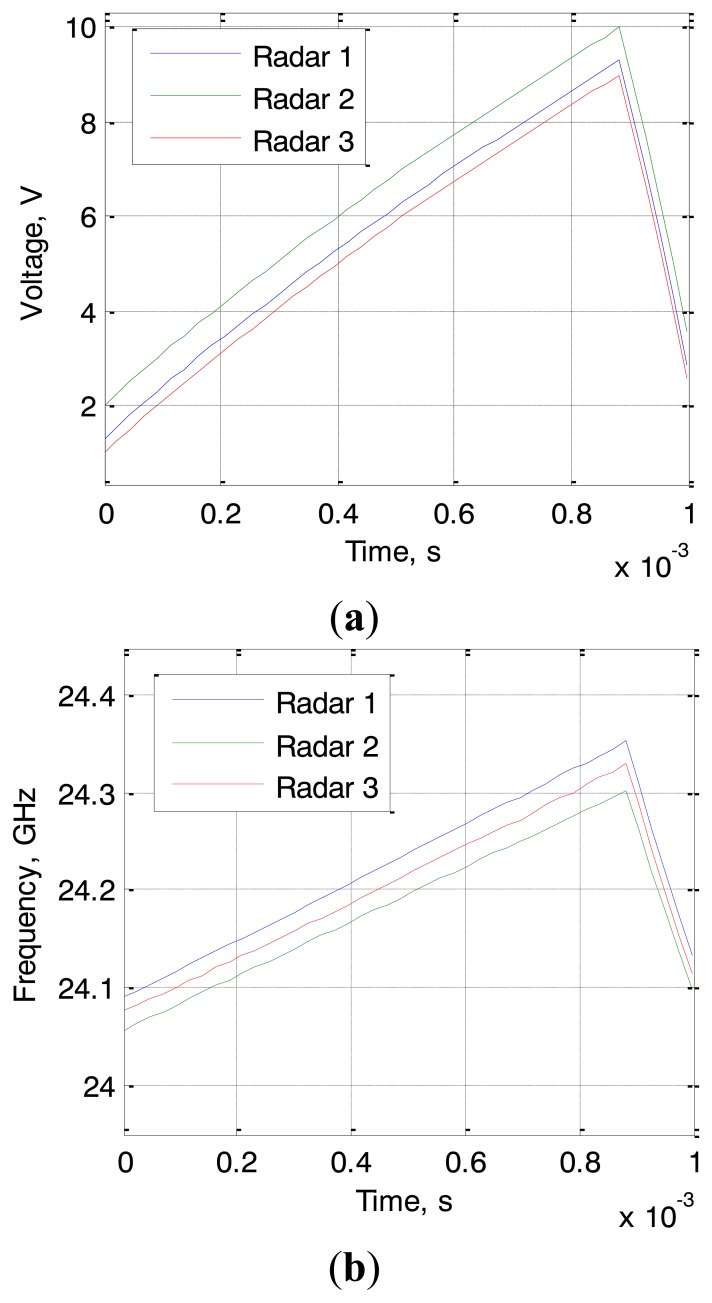
Configured OLFM-CW waveforms (**a**) Voltage ramps (**b**) Frequency ramps.

The radar network has been configured with OLFM-CW waveforms. The minimum frequency offset was configured at 12.5 MHz. This value is larger than the required by Equation (9) in order to accommodate frequency drifts and non-linearity of the VCOs. It must be taken into account that the VCOs of our three radars are configured in open-loop and their behaviors have different variation with the temperature. The voltage ramps that excite the VCOs and the resulting frequency ramps are illustrated in Figure 10.

**Figure 11.**
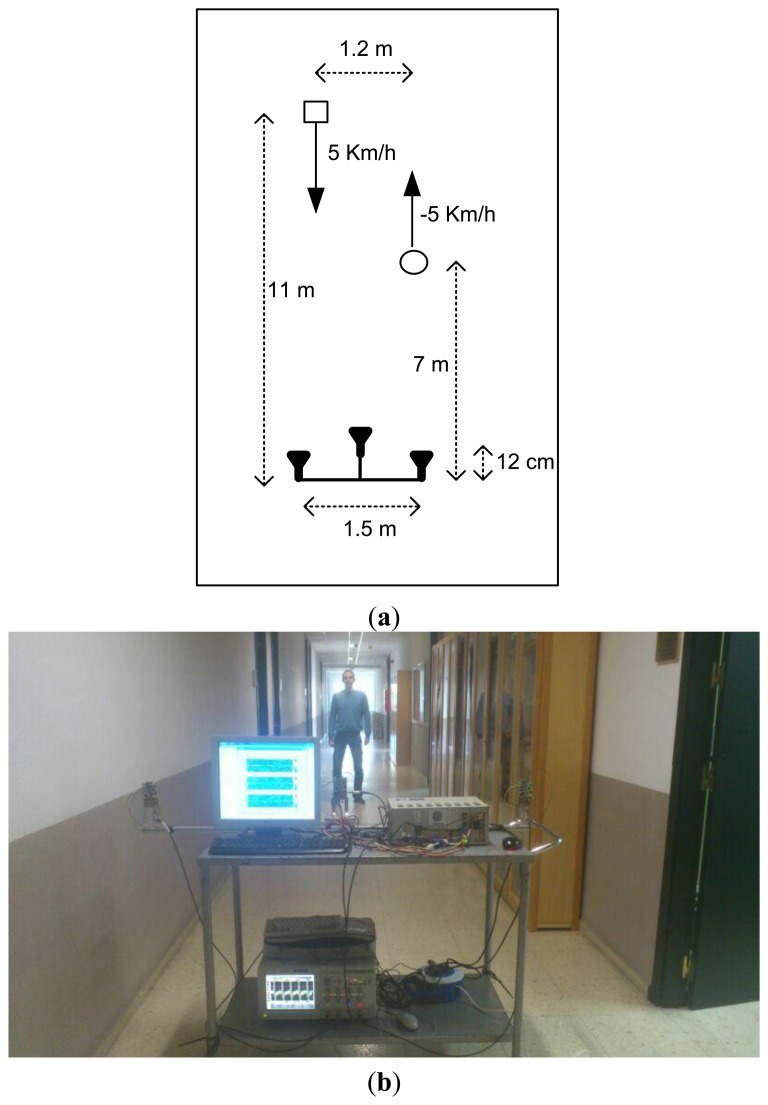
Experimental scenario (**a**) Top-view scheme (**b**) Photograph of the corridor.

To demonstrate the feasibility of the proposed system a real experiment has been carried out. The experiment consists of two people moving in front of the radar network in a corridor of our university. The experimental setup is shown in Figure 11.

The first person comes to the radar and the second one goes away the radar during five seconds of record. Both of them start to walk at the beginning of the record and carry on walking with a mean speed of 5 km/h.

**Figure 12.**
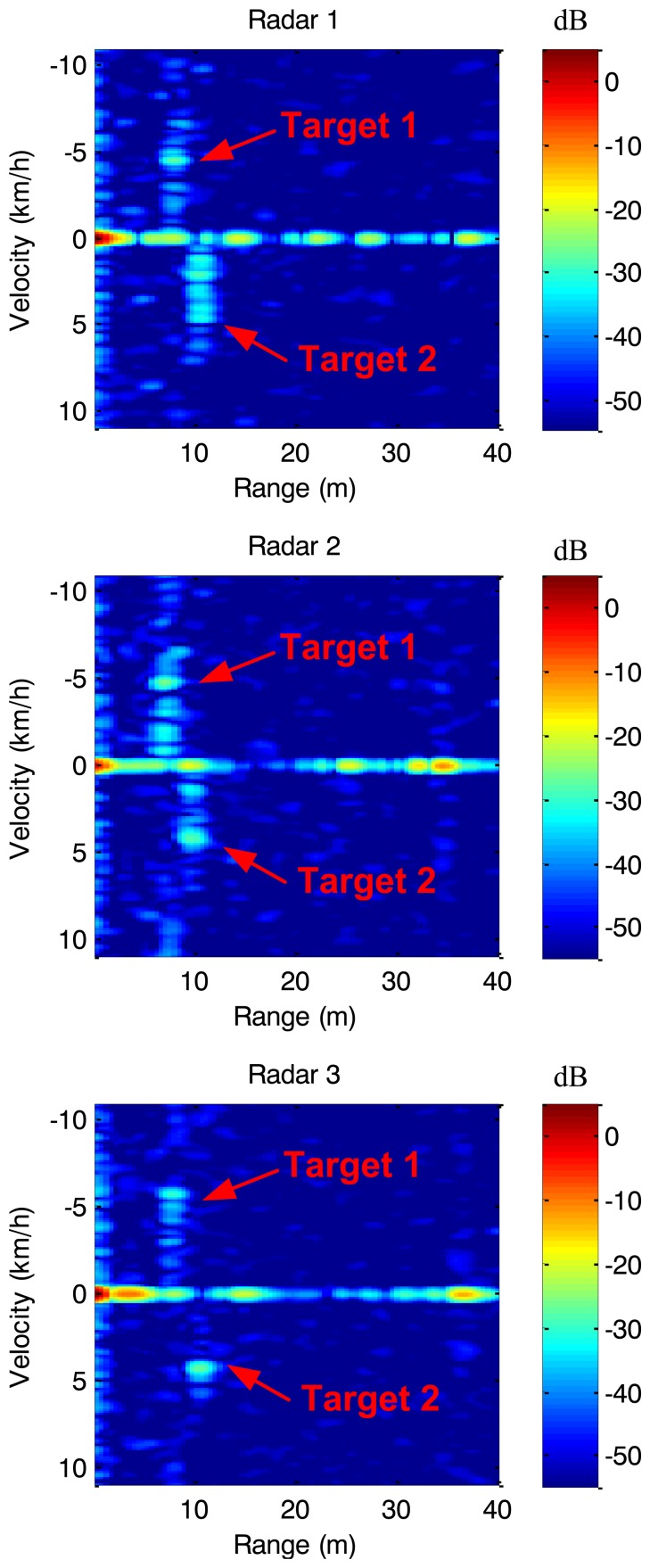
Range-Velocity maps of one frame of the record.

Figure 12 illustrates one frame corresponding to 100 ms of the record for each sensor. The range-velocity signatures of the targets have been highlighted. We can see the Doppler spreading of the two people due to the motion of arms and legs that complicates the detection task. To overcome this drawback an automatic detection procedure based on OSCA-CFAR has been implemented [11]. Furthermore, we can see the fixed clutter in the zero-velocity row of the images.

**Figure 13.**
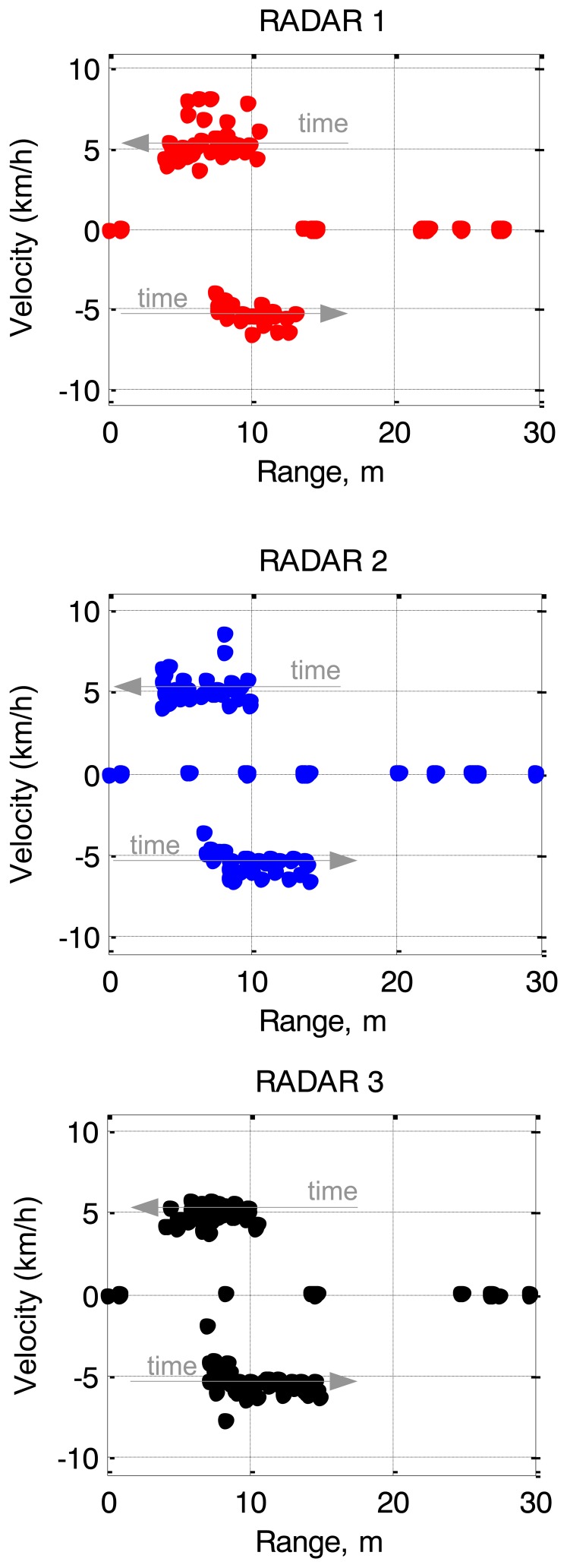
Detection map of the three sensors after OSCA-CFAR detector.

Figure 13 shows the results of the detection procedure during the five seconds of record in a velocity-range map for the three sensors of the network. Both pedestrians have been detected by the three sensors during the time of the recording. The speeds and positions during the recording are in agreement with the theoretical values.

There are several additional detections corresponding to fixed targets of the corridor and ground clutter. Also, there are some false alarms, e.g., the nearest detections due to the limited isolation between transmitter and receiver, and multiple detections of the two people due to their Doppler spreading.

Nowadays, we are working in the data fusion procedure. The future data fusion chain will remove false alarms thanks to the redundant information of the sensors. Furthermore, the data fusion algorithm will give azimuth information of the targets using multilateration techniques. Multilateration is based on calculating the intersection of *n* circles around the *n* sensors with the radius being the measured range. Several alternatives using minimum mean square error estimators have been proposed in the literature [3,9]. This angular information is very useful for the visualization subsystem.

## 5. Conclusions

This paper analyzes the problem of using a radar network as a collision warning system aboard a dumper. False alarm rate is a critical parameter for this application, due to the high cost of stopping a dumper often. False alarms due to ground clutter can be reduced using radar sensors with LFM waveforms. This is because LFM radars carry out the detection task over the range-Doppler domain, where a higher signal-to-clutter ratio can be achieved.

In the case of a radar network, bandwidth, power and time resources must be shared between all the sensors reducing self-interferences at the same time. This article proposes the use of OLFM-CW signals to overcome this problem. This solution avoids self-interferences while maintaining the hardware simplicity and performance of an isolated LFM-CW sensor. Finally, a radar network has been developed to demonstrate that the use of low-cost OLFM-CW radar transceivers in a collision warning application is a feasible alternative.

## Figures and Tables

**Table 1.** Radar network configuration.

**Parameter**	**Value**
Sweep bandwidth (*B_o_*)	240 MHz
Central frequency (*f_0_*)	24.2 GHz
Pulse repetition interval (*PRI*)	1.02 ms
Ascending ramp duration (*T*)	0.918 ms
IF bandwidth (*B_IF_*)	70 kHz
Waveform	OLFM-CW
Minimum frequency offset *(δ)*	12.5 MHz
Elevation beamwidth	12 deg
Azimutal beamwidth (*θ_a_*)	80 deg
CPI	100 ms
